# Development and validation of a quantitative electron microscopy score to assess acute cellular stress in the human exocrine pancreas

**DOI:** 10.1002/cjp2.185

**Published:** 2020-11-22

**Authors:** Nicole Kattner, Nicola Dyson, Yvonne Bury, Dina Tiniakos, Kathryn White, Tracey Davey, Lena Eliasson, Lynn Tindale, Bart E Wagner, Minna Honkanen‐Scott, Jennifer Doyle, Rutger J Ploeg, James AM Shaw, William E Scott

**Affiliations:** ^1^ Translational and Clinical Research Institute Newcastle University Newcastle upon Tyne UK; ^2^ Department of Cellular Pathology, Royal Victoria Infirmary Newcastle upon Tyne Hospitals NHS Foundation Trust Newcastle upon Tyne UK; ^3^ Department of Pathology, Aretaieion Hospital, Medical School National and Kapodistrian University of Athens Athens Greece; ^4^ Electron Microscopy Research Services Newcastle University Newcastle upon Tyne UK; ^5^ Department of Clinical Sciences Malmö, Islet Cell Exocytosis, Lund University Diabetes Centre Lund University Malmö Sweden; ^6^ Histopathology Department Royal Hallamshire Hospital Sheffield UK; ^7^ Nuffield Department of Surgical Science University of Oxford, BRC Oxford and NHS Blood and Transplant Oxford UK; ^8^ Institute of Transplantation, Freeman Hospital Newcastle upon Tyne Hospitals NHS Foundation Trust Newcastle upon Tyne UK

**Keywords:** ultrastructure, pancreas, transplantation, ischaemia, acute stress, histology

## Abstract

The pancreas is particularly sensitive to acute cellular stress, but this has been difficult to evaluate using light microscopy. Pancreatic ischaemia associated with deceased organ donation negatively impacts whole‐organ and isolated‐islet transplantation outcomes. *Post‐mortem* changes have also hampered accurate interpretation of *ante‐mortem* pancreatic pathology. A rigorous histological scoring system accurately quantifying ischaemia is required to experimentally evaluate innovations in organ preservation and to increase rigour in clinical/research evaluation of underlying pancreatic pathology. We developed and validated an unbiased electron microscopy (EM) score of acute pancreatic exocrine cellular stress in deceased organ donor cohorts (development [*n* = 28] and validation [*n* = 16]). Standardised assessment led to clearly described numerical scores (0–3) for nuclear, mitochondrial and endoplasmic reticulum (ER) morphology and intracellular vacuolisation; with a maximum (worst) aggregate total score of 12. In the Validation cohort, a trend towards higher scores was observed for tail versus head regions (nucleus score following donation after brainstem death [DBD]: head 0.67 ± 0.19; tail 0.86 ± 0.11; *p* = 0.027) and donation after circulatory death (DCD) versus DBD (mitochondrial score: DCD (head + tail) 2.59 ± 0.16; DBD (head + tail) 2.38 ± 0.21; *p* = 0.004). Significant mitochondrial changes were seen ubiquitously even with short cold ischaemia, whereas nuclear and vacuolisation changes remained mild even after prolonged ischaemia. ER score correlated with cold ischaemia time (CIT) following DBD (pancreatic tail region: *r* = 0.796; *p* = 0.018). No relationships between CIT and EM scores were observed following DCD. In conclusion, we have developed and validated a novel EM score providing standardised quantitative assessment of subcellular ultrastructural morphology in pancreatic acinar cells. This provides a robust novel tool for gold standard measurement of acute cellular stress in studies evaluating surrogate measures of peri‐transplant ischaemia, organ preservation technologies and in samples obtained for detailed pathological examination of underlying pancreatic pathology.

## Introduction

The pancreas is particularly susceptible to acute cellular injury. This is manifest in deceased donor pancreata procured for vascularised whole‐organ and isolated‐islet transplantation which are subject to *peri‐mortem* ischaemic and inflammatory stress associated with donation after brainstem death (DBD) or warm ischaemia associated with donation after circulatory death (DCD) [1, 2, 3, 4]. This is exacerbated by logistically necessary periods of cold ischaemia during organ storage prior to transplantation. Rates of conversion of a procured organ into a clinical transplant are low at 40% for DBD and 55% for DCD in the UK, with the majority of pancreata used for whole organ transplantation [5, 6].

Cold ischaemic time (CIT) is an independent predictor of 90‐day vascularised pancreas graft survival and associations with post‐transplant graft pancreatitis, thrombosis and technical failure have been reported [7, 8, 9]. The additional warm ischaemic time (WIT) in DCD pancreata is associated with more frequent occurrence of complications including graft thrombosis [4]. Early complications, graft rejection and technical graft failure of pancreas transplantation are associated with damage/stress in the exocrine compartment comprising up to 95% of the pancreas [4, 9, 10]. Furthermore, graft rejection episodes can impact on long‐term function or lead to graft loss with the diagnosis remaining challenging [8, 11].

Isolation of islets from a donor pancreas provides a minimally invasive alternative to whole pancreas transplantation but yields remain relatively limited and are negatively impacted by CIT [12, 13]. Normal functional β‐cell mass is not restored in the recipient and long‐term insulin independence is not routinely achieved [14, 15]. Increasing CIT is associated with poorer transplantation outcomes [7]. Within the unified Pancreas Allocation Scheme in the UK providing equity of access to recipients, 16% of DBD β‐cell (pancreas and islet) transplants and 7% of DCD β‐cell transplants were as isolated islets in 2018–2019 [5, 6, 7, 12].

Pancreatic donor risk scores integrating the multiple parameters which may induce acute pancreatic cellular stress and impact transplant outcomes have been developed into evidence‐based clinical decision‐making tools. However, the predictive value of these indices remains imperfect [16, 17, 18, 19]. None of these have been validated by gold standard pathological assessment of the donor organ.

Enhanced pancreas preservation technologies including normothermic regional perfusion, hypothermic/normothermic *ex vivo* perfusion, and persufflation are being innovated towards prevention and reversal of acute cellular stress [20, 21, 22, 23, 24, 25, 26, 27]. Assessment of the impact of these interventions on acute stress at a cellular level has largely been limited to qualitative light microscopy of standard histological stains, precluding quantitative assessment of severity [23, 24].

The extreme sensitivity of the pancreas to ischaemia may also lead to misinterpretation of underlying histopathology in resected tissue, particularly when procured *post‐mortem*. Previously reported absence of substantial β‐cell proliferation in the adult pancreas has been questioned following evidence of rapid loss of Ki67 staining positivity over a period of cold preservation [28]. A robust score of overall ischaemic impact on the organ would enable better informed distinction of *post‐mortem* artefact from pre‐existing pathology.

Our aims were to develop an unbiased transmission electron microscopy (TEM) quantitative measure of acinar cell stress at an ultrastructural level in a cohort of deceased donor pancreata and to validate this in parallel with conventional light microscopy evaluation in a second cohort.

## Materials and methods

### Pancreas donors, procurement and shipment

All studies were performed under specific ethical approvals (Research Ethics Committee for Wales) and written donor‐relative consent in compliance with the UK Human Tissue Act of 2004. Procurement of more recent organs (including all within the Validation cohort) was subject to additional ethical approval as a Blood and Transplant Research Unit study which was approved by the UK Human Research Authority and reviewed by the NRES Committee North East (Newcastle & North Tyneside 1: 16NE0230).

Pancreata not placed for clinical transplantation were procured from deceased organ donors as if for transplant by the UK National Organ Retrieval Service using standardised protocols [29]. In brief, whole pancreata were flushed with University of Wisconsin (UW) cold storage solution *in situ*, and packaged in three concentric bags; with UW between each bag layer. The pancreas was then generously covered with ice in an insulated box for shipment to Newcastle University by a licensed courier.

### Biopsy collection and staining

Upon arrival, the pancreas was carefully removed from its packaging and placed into a pan containing UW solution at 4 °C for dissection. Prior to commencement of the Quality in Organ Donation (QUOD) MRC Expand vascularised pancreas biobanking initiative, samples were collected from uncinate process, head, body and tail. Within the QUOD initiative (which included all organs within the Validation cohort), protocols were further refined with all dissection and sampling performed inside a cold room at 4 °C to avoid unintended tissue warming due to handling. Briefly, spleen and duodenum were carefully dissected off followed by removal of surrounding adipose tissue and blood vessels. The pancreas was divided into eight regions (P1–P8), with P1 corresponding to the head/uncinate process and subsequent blocks moving incrementally towards the tail region (P8).

To obtain samples for histological assessment, biopsies with maximum thickness of 5 mm in a single dimension were collected from each region and fixed in 10% Neutral Buffered Formalin (Sigma Aldrich, Gillingham, UK or CellPath Ltd., Newtown, UK). Further processing including dehydration, clearing, wax infiltration followed by embedding in paraffin, section cutting, and tissue staining with a Discovery Ultra system (Ventana Bioscience, San Diego, CA, USA) was performed by the Cellular Pathology Node laboratory at the Royal Victoria Infirmary, Newcastle upon Tyne.

To obtain samples for ultrastructural assessment, small tissue pieces were cut, stained with dithizone to visualise islets under a dissecting microscope, further dissected into 1–2 mm^3^ biopsies including visible islets, and fixed in 2% glutaraldehyde 0.1 M cacodylate buffer (Agar Scientific, Essex, UK). Islet enrichment was undertaken to enable EM studies of the pancreatic endocrine compartment, although the current studies focused on the predominant exocrine compartment to ensure sufficient cell numbers for representative sampling. It was separately confirmed that dithizone staining did not artefactually impact EM (sub)cellular morphological appearances (data not shown).

Early samples were prepared for TEM using a heavy metal protocol with later samples including all within the Validation cohort prepared using a standard EM protocol. Samples were embedded in epoxy resin (TAAB, Aldermaston, UK) before assessment of 70 nm ultrathin sections. Biopsies processed according to the standard EM protocol were subsequently stained with uranyl and lead. Samples were processed by the Electron Microscopy Research Services at Newcastle University.

### Histological and ultrastructural assessment of pancreas biopsies

Assessment was performed on two separate samples from a corresponding area biopsied in routine sizes for each protocol. Semiquantitative histological assessment of H&E and Sirius Red/Fast Green (SRFG)‐stained routine sections for pre‐existing pathology and acute changes was performed by a consultant clinical histopathologist (YB) with expertise in pancreas pathology. Stained sections were available as standard glass slides and digitised. For ultrastructural assessment, ultrathin sections were placed on Ni‐grids with a chloroform film and analysed with the Philips CM100 (Philips Electron Optics, Eindhoven, Netherlands) or the Hitachi HT7800 120 kV (Hitachi High‐Technologies, Maidenhead, UK) TEM by trained personnel (ND, NK).

### Statistics

Data are reported as mean (range). Means were compared by independent sample and paired Student's *t*‐tests performed using IBM SPSS® statistics v25 (IBM, Portsmouth, UK). Relationships between donor parameters and EM scores were assessed by two‐tailed Pearson correlation coefficients calculated using IBM SPSS® statistics v25. Graphs were created using GraphPad Prism v8 (GraphPad, San Diego, CA, USA).

## Results

### Evaluation of range of normality, established pathology and acute stress at an EM ultrastructural level

Detailed analysis of TEM appearances in biopsies from the Development cohort of 28 donor pancreata was undertaken. Blinded assessors evaluated normal sub‐cellular morphological appearances and observed for the spectrum of changes associated with acute stress in acinar cells. Mean age was 53 years and donors with a wide range of age (25–74 years), body mass index (BMI) (17.7–34.9) kg/m^2^ and CIT (4.2–38.1 h) were evaluated. 54% were female and 49% were DCD with mean (range) of functional warm ischaemia [30] of 25 (12–34) minutes (see supplementary material, Table S1).

**Figure 1:**
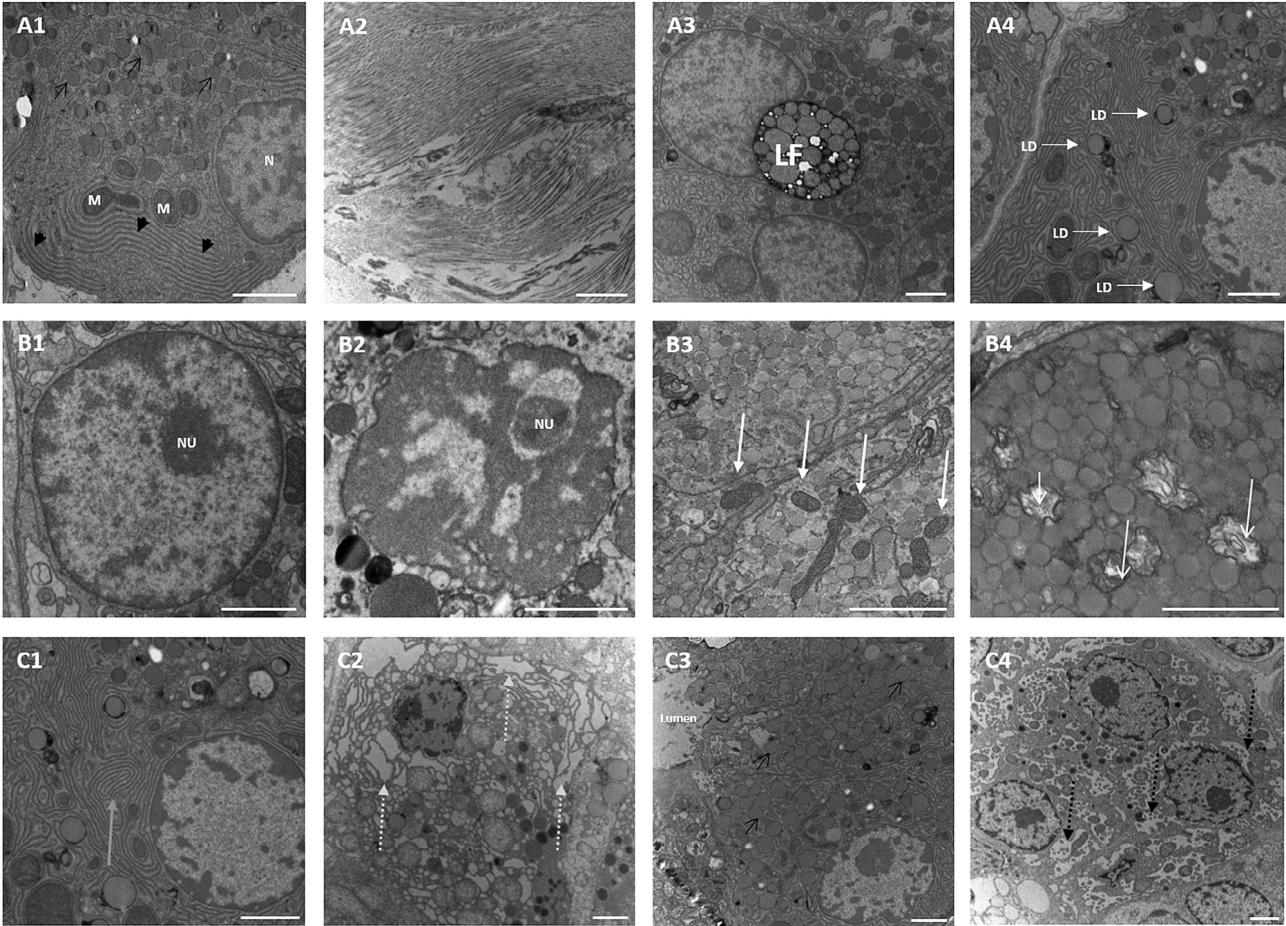
TEM analysis of human pancreatic acinar cells. (A1) Acinar cell with nucleus (N), mitochondria (M), ER (thick black arrows) and characteristic acinar vesicles (thin black arrows). Pre‐existing pathology with collagen fibres in the stroma (A2), lipofuscin (LF) (A3) and intracellular lipid droplets (LD, white arrows) (A4). Impact of acute stress on acinar morphology with (B1) normal nucleus with dispersed chromatin and nucleolus (NU), (B2) clumped chromatin with nucleolus (NU), (B3) normal mitochondria (white thick arrows), (B4) swollen mitochondria with destroyed morphology and cristae (white thin arrows), (C1) normal ER (grey arrow), (C2) dilated ER (dashed grey arrows), (C3) normal acinar tissue morphology with acinar cells identified by characteristic vesicles (thin black arrows) grouped around a lumen, and (C4) development of vacuoles (dashed black arrows) with increasing impact on morphology. Images derived from the Development cohort (see supplementary material, Table S1). Scale bars: 2 μm.

The range of normal sub‐cellular organelle morphologies in the pancreatic exocrine compartment was determined through systematic examination of individual cells. Normal acinar cells could be identified by cytoplasmic acinar vesicles (thin black arrows) and extensive presence of endoplasmic reticulum (ER) (black arrowheads) (Figure 1A1). The nucleus (N) and mitochondria (M) were also visible.

Underlying pathologies including stromal collagen fibres, lipofuscin (LF) and lipid droplets (LD) were observed at an ultrastructural level (Figure 1A2–A4). Depending on their orientation, collagen fibres appeared as electron dense lines or circles and accumulated in the stroma surrounding the cells. LF pigment was identified as cytoplasmic black circular bodies of variable size containing several less electron dense rings of variable diameter. Additionally, cytoplasmic LD were present in the cytoplasm appearing as low electron‐density circles with a high electron‐dense periphery.

A spectrum of acute changes at the ultrastructural level were observed in different organelles and, iteratively, a standardised approach of evaluating nucleus, mitochondria and ER, as well as changes in overall cell morphology due to formation of vacuoles was developed:

#### Nucleus

Normal nuclei possessed a dark double layered electron dense membrane containing chromatin which had higher electron‐density and was distributed evenly through the whole nucleus (Figure 1B1). In some cases, a nucleolus could be observed. Following acute stress, some nuclei displayed chromatin clumping or detachment from the nuclear membrane (Figure 1B2).

#### Mitochondria

Mitochondria normally contain a double‐layer membrane appearing as electron‐dense lines, with the inner layer building characteristic cristae (Figure 1B3, white arrows). In most cases, several mitochondria were observed within a cell. Acute stress on mitochondria resulted in altered morphology with either a more open or a swollen appearance, whilst the grade of swelling varied from mild to severe. In some instances, the cristae were either altered or completely destroyed (Figure 1B4, white arrows).

#### Endoplasmic reticulum

Normal ER is visualised as electron‐dense parallel lines distributed in large areas within the cytoplasm (Figure 1C1, grey arrow). Acute changes to ER resulted in diffuse dilation, appearing as low electron‐density regions between darker electron‐dense lines (Figure 1C2, grey arrows). Increasing dilation impacts on cell morphology, which impaired clear identification of cell margins and organelles.

#### Vacuolisation

Acute stress resulted in cell vacuolisation with cytoplasmic vacuoles appearing as circular light electron‐dense areas surrounded by a dark electron‐dense membrane (Figure 1C4, black arrows). Variable vacuole size altered acinar cell morphology (Figure 1C3).

### Development of a standardised numerical scoring system to assess acute stress in pancreatic acinar cells

The Newcastle Pancreatic Acinar Stress Score (NPASS) was developed based on the above ultrastructural observations to assess changes to overall cell and cell‐organelle morphology of acinar cells to enable an unbiased evaluation of the severity of ischaemic injury impact and donated tissue quality. The status of nuclei, mitochondria, ER and vacuolisation were assessed on a 4‐point scale with the sum of these generating an aggregate total score. Individual NPASS for each feature ranged from 0 to 3, with 0 indicating normal morphology and 3 worst degradation through acute injury yielding a possible range of 0–12 in total score (Table 1).

**Table 1:** NPASS criteria.

Score	Nucleus	Mitochondria	ER	Vacuolisation
0	Normal appearance	Normal appearance	Normal appearance	Absent
1	Chromatin condensation (mild–moderate)	Open appearance	Mild dilation	Mild vacuolisation (0–25% of cell)
2	Chromatin condensation (moderate–severe)	Swollen appearance	Moderate dilation	Moderate vacuolisation (25–50% of cell)
3	Chromatin condensation and detachment from nuclear membrane	Swollen appearance and disrupted cristae	Severe dilation	Severe vacuolisation (>50% of cell)

Acinar cells on sections from both the head and tail of pancreata within the Development cohort were examined. To avoid bias of cell selection for assessment and to ensure a representative score for the whole sections, five areas across the section were defined: one in the centre of the section and four in each of the surrounding quadrants. Images were captured at low magnification (×1500) for each area.

From within these five areas, five acinar cells were selected at random for analysis, and higher magnification images of each cell were taken for subsequent scoring. Images were captured at a minimum magnification of ×4000 for assessment of nuclei, ER and vacuolisation; and a minimum of ×6000 was set for mitochondria.

Mean score from 25 individual acinar cells within each sample was calculated for each individual parameter as well as for the total score. Scoring was performed by two individuals (observer 1 [NK] and observer 2 [ND]) and mean values were reported. Data was normally distributed.

### Histological scoring of a Validation cohort of donor pancreata

The Validation cohort comprised 16 pancreata with 7 (44%) female donors. Mean (range) age was 48.9 (24–71) years and BMI 27.0 (21.8–37.2) kg/m^2^. CIT was 12.6 (3.6–25.6) h. 50% were DCD with functional WIT 17.7 (0–103) minutes. Additional donor data including known diabetes or pre‐admission history of out‐of‐hospital cardiopulmonary arrest, length of intensive therapy unit (ITU) stay and cause of death are included in Table 2.

**Table 2:** Donor information and NPASS in the Validation cohort.

											NPASS									
Donor data											Head					Tail				
Donor no	CIT (h)	WIT (min)	Donor type	Age (years)	Gender	BMI (kg/m^2^)	ITU stay (days)	Out of hospital cardiac arrest	Cause of death	History of diabetes	Nucleus	Mitochondria	ER	Vacuolisation	Total score	Nucleus	Mitochondria	ER	Vacuolisation	Total score
DBD‐1	3.6	–	DBD	71	F	26.3	1	No	ICH	No	0.82	2.27	1.22	1.06	5.37	0.86	2.51	1.56	1.00	5.93
DBD‐2	4.2	–	DBD	67	M	22.9	13	No	ICH	No	0.54	2.09	1.40	1.22	5.25	0.64	2.34	1.54	1.16	5.63
DBD‐3	7.7	–	DBD	60	F	26.7	2	No	ICH	T1D	1.06	2.41	0.78	0.84	5.09	0.90	2.51	1.78	1.28	6.47
DBD‐4	10.5	–	DBD	71	F	30.9	2	No	ICH	No	0.52	2.12	2.17	1.10	5.86	0.84	2.25	1.72	1.12	5.93
DBD‐5	15.5	–	DBD	27	F	28.2	2	No	ICH	No	0.66	2.63	2.16	1.22	6.67	0.88	2.63	1.54	1.00	6.05
DBD‐6	19.6	–	DBD	25	M	25.0	3	Yes	Overdose	No	0.48	2.43	1.32	0.66	4.89	0.84	2.44	2.14	1.04	6.46
DBD‐7	24.7	–	DBD	47	F	21.8	2	No	ICH	No	0.70	2.03	2.36	1.62	6.71	0.90	2.19	2.04	1.24	6.37
DBD‐8	25.6	–	DBD	71	F	24.8	2	No	ICH	No	0.60	2.74	1.56	0.82	5.72	1.02	2.45	2.00	1.04	6.51
Mean	13.9	–	–	55	–	25.8	3	–	–	–	0.67	2.34	1.62	1.07	5.70	0.86	2.42	1.79	1.11	6.17
Range	3.6–25.6	–	–	25–71	–	21.8–30.9	1–13	–	–	–	0.48–1.06	2.03–2.74	0.78–2.36	0.66–1.62	4.89–6.71	0.64–1.02	2.19–2.63	1.54–2.14	1.00–1.28	5.63–6.51
Summary	–	–	–	–	6 (75%) female	–	–	1 (12.5%) OHCA	7 (87.5%) ICH	7 (87.5%) non‐diabetic	–	–	–	–	–	–	–	–	–	–
DCD‐1	4.4	28	DCD	52	M	29.4	17	No	Respiratory failure	T2D	0.60	2.63	1.90	0.96	6.09	0.56	2.43	2.64	1.98	7.61
DCD‐2	6.3	25	DCD	60	F	23.6	3	No	ICH	No	0.70	2.59	1.56	0.82	5.67	0.92	2.77	1.94	1.30	6.93
DCD‐3	7.8	33	DCD	38	M	29.1	3	No	ICH	T2D	0.90	2.66	2.20	1.32	7.08	1.34	2.32	2.08	1.34	7.08
DCD‐4	8.4	25	DCD	62	M	26.9	2	No	ICH	No	0.92	2.63	1.22	1.22	5.87	0.92	2.44	1.54	0.80	5.70
DCD‐5	12.9	31	DCD	24	M	27.8	3	Yes	Asphyxiation	No	0.38	2.28	2.58	1.24	6.48	0.76	2.75	1.96	1.14	6.61
DCD‐6	12.9	103	DCD	36	M	27.8	16	Yes	Overdose, asphyxiation	No	0.84	2.59	1.80	1.30	6.53	0.64	2.87	0.74	0.62	4.87
DCD‐7	15.1	n/a	DCD	45	M	24.6	n/a	n/a	n/a	n/a	0.88	2.70	1.14	0.76	5.48	0.58	2.54	1.62	1.00	5.74
DCD‐8	22.8	20	DCD	27	M	37.2	4	No	ICH	No	0.65	2.68	1.80	1.40	6.48	1.12	2.49	2.70	1.54	7.85
Mean	11.3	38	–	43	–	28.3	7	–	–	–	0.73	2.60	1.78	1.13	6.21	0.86	2.58	1.90	1.22	6.55
Range	4.4–22.8	20–103	–	24–62	–	23.6–37.2	2–17	–	–	–	0.38–0.92	2.28–2.70	1.14–2.58	0.76–1.40	5.48–7.08	0.56–1.34	2.32–2.87	0.74–2.70	0.62–1.98	4.87–7.85
Summary	–	–	–	–	1 (12.5%) female	–	–	2 (25%) OHCA	4 (50%) ICH	5 (62.5%) non diabetic	–	–	–	–	–	–	–	–	–	–

Clinical data and NPASS values in eight DBD and eight DCD ordered by ascending CIT, including WIT in minutes, donor type, age in years, gender (F, female; M, male), BMI in kg/m^2^, length of ITU stay in days, out of hospital cardiac arrest (OHCA), cause of death (ICH‐intracranial haemorrhage), and history of diabetes (T1D, type 1 diabetes; T2D, type 2 diabetes). Right section shows mean NPASS values (from 25 analysed cells) for nuclei, mitochondria, ER, vacuolisation and total score for head and tail region in each donor pancreas.

**Figure 2:**
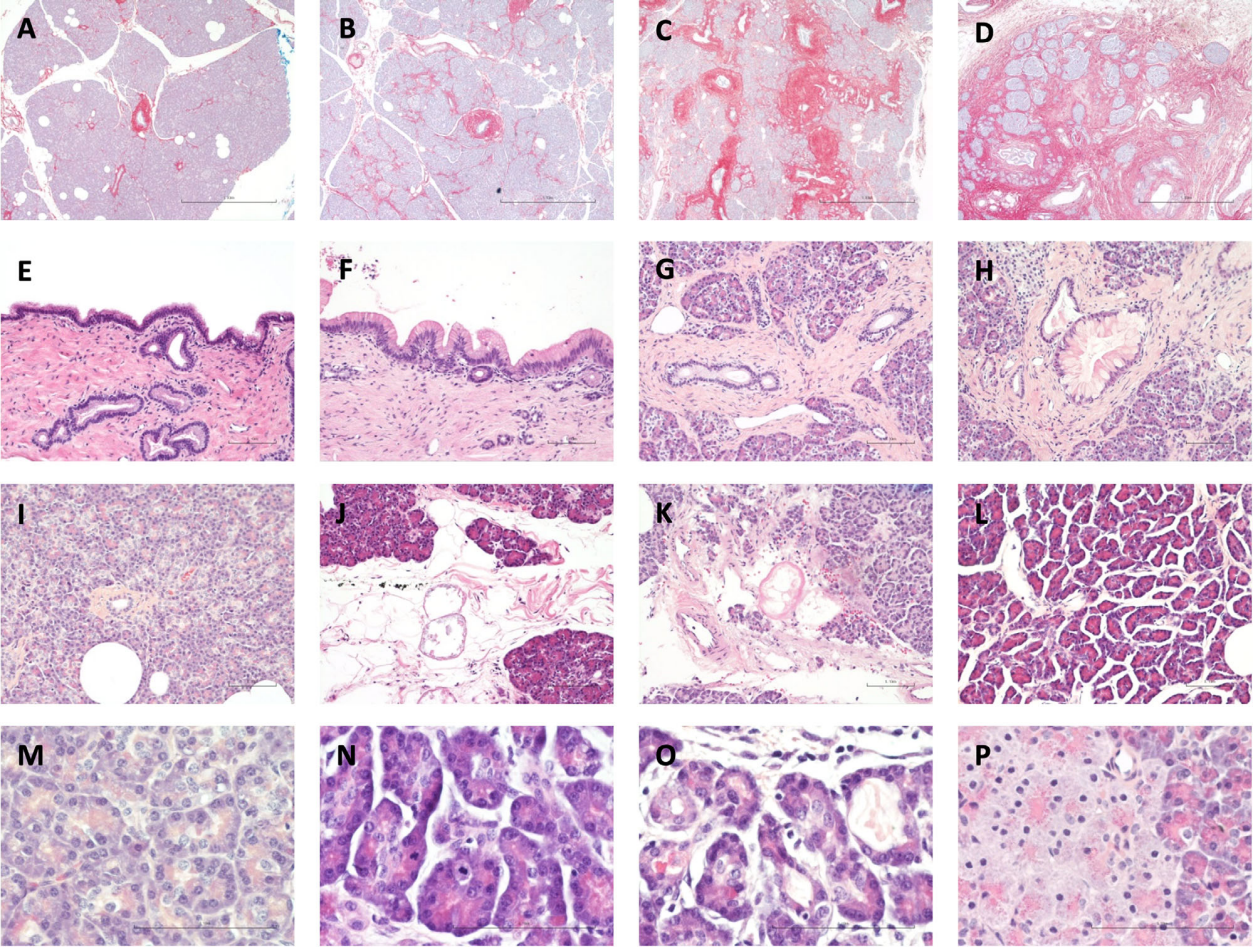
Histological analysis of donor human pancreas. (A) DCD‐2, head: negligible fibrosis, (B) DBD‐7, head: mild intra‐lobular fibrosis, (C) DBD‐3, tail: moderate peri‐ductal and intra‐lobular fibrosis, (D) DBD‐8, tail: severe fibrosis with atrophy of the exocrine compartment and clustering of residual islets (A–D: Sirius Red Fast Green ×40), (E) DBD‐5, head: normal duct epithelium in the main pancreatic duct, (F) DBD‐8, head: low grade PanIN in the main pancreatic duct, (G) DBD‐7, tail: normal branch duct epithelium, (H) DBD‐7, tail: low grade PanIN in branch duct epithelium, (I) DCD‐8, head: normal exocrine compartment showing good preservation with most acini consisting of spherically arranged individual acinar cells, (J) DBD‐7, head: focal fat necrosis, (K) DCD‐8, head: focal fat necrosis, (L) DBD‐5, tail: interstitial oedema (E–L: H&E, ×20), (M) DCD‐8, head: normal exocrine compartment showing good preservation of cell structure with abundant granular eosinophilic cytoplasm in the apical aspect consistent with intact zymogen granules, with basophilic basal cytoplasm, (N) DCD‐6, head: mitotic activity in acini in an area of mild interstitial oedema, (O) DCD‐6, head: acinar ectasia and degenerative changes of the acinar epithelium with flattening in an area of mild interstitial oedema, (P) DBD‐1, head: acinar nodule or ischaemic focus with shrunken nuclei with condensed chromatin, pallor of the acinar cytoplasm, and residual intact apical zymogen granules (M–P: H&E ×600).

Blocks from head (P1) and tail (P8) of each donor pancreas were assessed twice, 2 months apart by a single pathologist (YB). The first assessment was blinded to clinical details. Histological assessment revealed pre‐existing pathology where present; including fibrosis (Figure 2A–D), low grade pancreatic intraepithelial neoplasia (PanIN) [31] (Figure 2E‐H), islet peliosis [32] (see supplementary material, Figure S1) as well as more acute changes including fat necrosis (Figure 2J,K), interstitial oedema (Figure 2L,N,O), and in a few cases eosinophilic acinar nodule formation (Figure 2P). Normal pancreatic exocrine histology is illustrated in (Figure 2I,M) for comparison.

Brief histopathology reports were compiled for all examined blocks (see supplementary material, Table S2) with a short summary in Table 3. None showed any incontrovertibly significant evidence of ischaemia. Features in keeping with possible minor acute cell injury were seen in 13 (41%) sections (Table 3). Presence of acinar cell nodules was observed in 5 (16%) sections. No severe fat necrosis, haemorrhagic necrosis or significant autolytic changes were observed. Low grade PanIN was observed in 7 (22%) of the analysed sections. Presence of fibrosis, classified as negligible, mild, moderate or marked was determined (Table 3). No associations between pathologist assessment and CIT; DBD versus DCD or other ischaemic risk factors such as death by asphyxiation or out of hospital cardiopulmonary arrest were evident.

**Table 3:** Histological assessment of donor pancreata in the Validation cohort with light microscopy.

	Head				Tail			
Donor number	PanIN	Fibrosis	Acinar cell nodules	Potential acute cell injury	PanIN	Fibrosis	Acinar cell nodules	Potential acute cell injury
DBD‐1	Absent	Negligible	Present (*n* = 1)	Absent	Absent	Negligible	Present (*n* = 3)	Absent
DBD‐2	Absent	Negligible	Absent	Possible very minor with few cells showing cytoplasmic pallor	Absent	Negligible	Absent	Possible very minor with few cells showing cytoplasmic pallor
DBD‐3	Absent	Mild	Absent	Possible very minor	Absent	Moderate/marked	Absent	Absent
DBD‐4	Present (low grade/1A)	Negligible	Absent	Absent	Absent	Negligible	Absent	Absent
DBD‐5	Absent	Mild/moderate	Absent	Absent	Absent	Mild/moderate	Absent	Possible very minor
DBD‐6	Absent	Mild	Absent	Absent	Absent	Mild/moderate	Absent	Possibly very minor
DBD‐7	Absent	Mild	Absent	Possible very minor with few cells showing cytoplasmic pallor	Present (low grade/1A)	Mild	Present (*n* = 2)	Possible
DBD‐8	Present (low grade/1A)	Mild	Absent	Possible very minor	Present (low grade/1A)	Marked	Absent	Absent
DCD‐1	Absent	Mild	Present (*n* = 15)	Absent	Absent	Mild	Present	Absent
DCD‐2	Absent	Negligible	Absent	Absent	Present (low grade/1A)	Negligible	Absent	Absent
DCD‐3	Present (low‐grade/1B)	Moderate	Absent	Absent	Absent	Moderate	No acinar cells or exocrine component seen/absent	Absent
DCD‐4	Absent	Marked	Absent	Absent	Present (low grade/1A)	Marked	Absent	Absent
DCD‐5	Absent	Negligible	Absent	Possible very minor	Absent	Negligible	Absent	Possible very minor
DCD‐6	Absent	Mild	Absent	Absent	Absent	Negligible	Absent	Possible very minor
DCD‐7	Absent	Mild	Absent	Absent	Absent	Negligible	Absent	Absent
DCD‐8	Absent	Negligible	Absent	Possible very minor	Absent	Negligible	Absent	Possible very minor

### Electron microscopy stress scores in the Validation cohort

For NPASS assessment, images of 25 acinar cells were captured by a trained operator (ND) within both the head and tail regions of the 16 pancreata of the Validation cohort. These images were used for independent scoring by two trained observers (NK and ND) whereby the mean score was calculated for each parameter and total score by combining the values of both scorers (Table 2).

**Figure 3:**
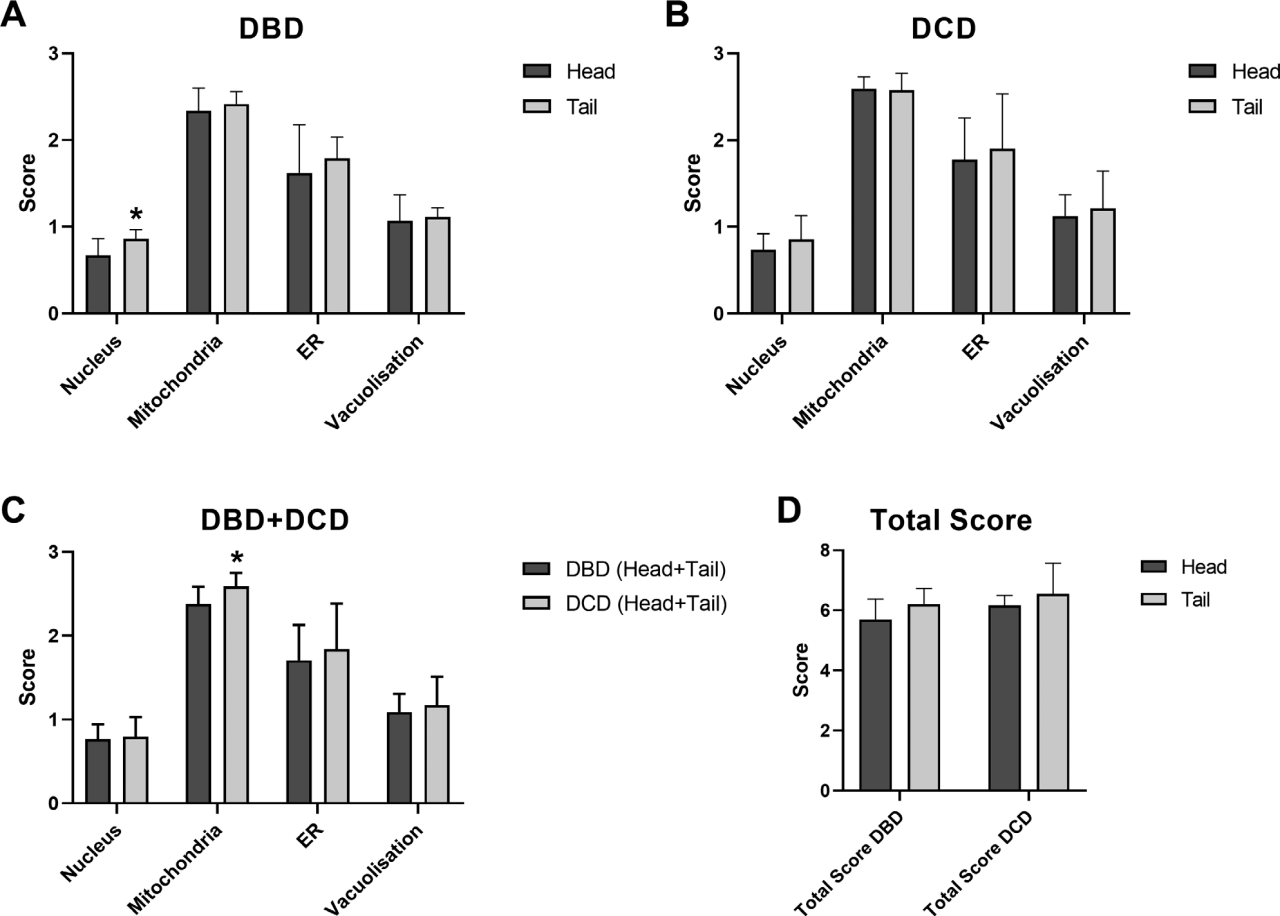
(A) Mean (+SD) nuclear, mitochondria, ER, vacuolisation and total NPASS values in head and tail regions of eight DBD pancreata. No significant difference was observed between paired head and tail scores for mitochondria (*p* = 0.254), ER (*p* = 0.448) and vacuolisation (*p* = 0.687). Mean nuclear score was significantly higher in the tail (*p* = 0.027). *p < 0.05 versus head. (B) Mean (+SD) NPASS values in head and tail regions of eight DCD pancreata. No significant difference was observed between the head and tail for nucleus (*p* = 0.288), mitochondria (*p* = 0.872), ER (*p* = 0.610), and vacuolisation (*p* = 0.652). (C) Mean NPASS (+SD) from eight DBD and eight DCD (head and tail regions). No significant difference was observed between scores of DBD and DCD for nuclei (*p* = 0.706), ER (*p* = 0.446) and vacuolisation (*p* = 0.422). Significantly higher NPASS scores were observed for mitochondria in DCD organs (*p* = 0.004). *p < 0.05 versus DBD. (D) Mean (+SD) total NPASS values of head and tail regions in eight DBD and eight DCD pancreata. No significant difference was observed between paired head and tail values of total score in DBD (*p* = 0.125) and DCD (*p* = 0.389).

In both DBD (Figure 3A,D) and DCD (Figure 3B,D) donors, there was a trend towards higher NPASS values (individual parameters and total) in the tail compared with the head of the pancreas. Comparative differences reached statistical significance for nucleus score in DBD (*p* = 0.027). Comparison of DBD with DCD pancreata (Figure 3C,D), showed trends towards higher scores in DCD, with significantly higher impact on mitochondria (*p* = 0.004).

**Figure 4:**
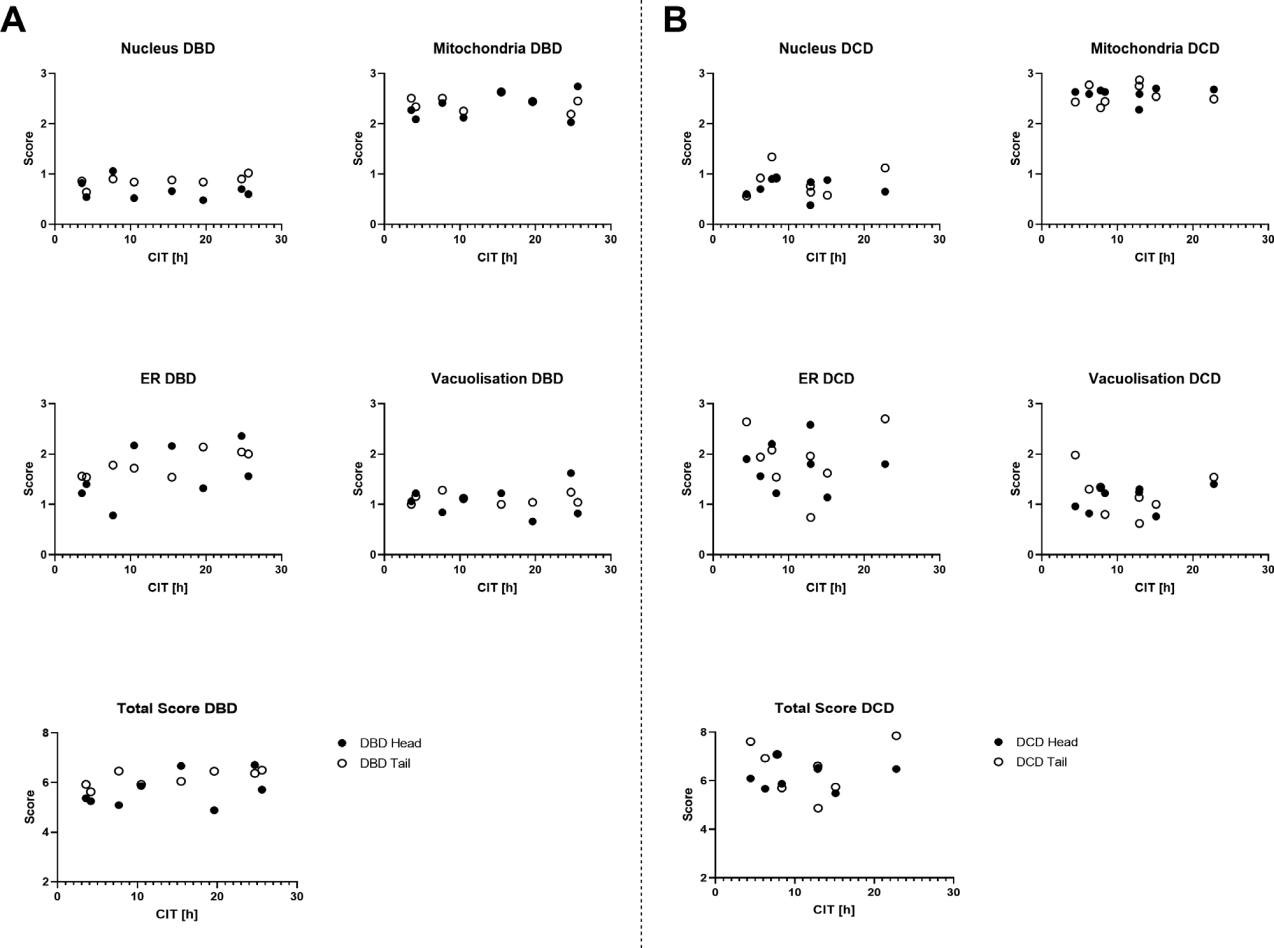
Correlation of NPASS values with donor CIT. Scores in head (black) and tail (white) region of each DBD (A) and DCD (B) pancreas for each NPASS parameter are plotted against CIT for that organ.

Scores for each parameter in addition to total scores for head and tail regions were plotted against CIT in DBD (Figure 4A) and DCD (Figure 4B) donors. Pearson's correlation coefficients are shown in Table 4. Nuclear chromatin condensation and vacuolisation scores remained relatively low even after prolonged ischaemic exposure. In contrast, mitochondrial morphological changes were marked even with short CIT. In DBD donors, ER dilation increased incrementally as CIT became longer, with most significant correlation in the pancreatic tail (Table 4). This was mirrored in the relationship between CIT and total score in DBD donors with highest correlation in the tail region (*r* = 0.710; *p* = 0.045). No correlations were observed between any of the NPASS parameters and CIT for DCD donors and no associations with warm ischaemia or other potential factors associated with significant ischaemia could be discerned.

**Table 4:** Correlation between NPASS values and CIT in the Validation cohort.

	DBD all		DBD head		DBD tail		DCD all		DCD head		DCD tail	
	Correlation coefficient	*P* value	Correlation coefficient	*P* value	Correlation coefficient	*P* value	Correlation coefficient	*P* value	Correlation coefficient	*P* value	Correlation coefficient	*P* value
Nucleus	0.017	0.950	−0.322	0.436	0.642	0.086	0.022	0.935	−0.107	0.801	0.112	0.791
Mitochondria	0.151	0.577	0.363	0.377	−0.202	0.632	0.092	0.734	0.059	0.889	0.118	0.781
ER	0.519	0.039	0.470	0.239	0.796	0.018	0.021	0.939	−0.024	0.955	0.055	0.896
Vacuolisation	0.016	0.954	0.047	0.912	−0.064	0.879	0.029	0.916	0.387	0.344	−0.173	0.681
Total score	0.455	0.077	0.450	0.264	0.710	0.048	0.048	0.860	0.125	0.769	0.014	0.974

## Discussion

Pancreatic assessment prior to transplantation remains challenging, with inadequate understanding of the impact of donor factors and peri‐transplant tissue handling on acute cellular stress within the organ. We have developed and validated an electron microscopy scoring system enabling robust and unbiased evaluation of (sub)cellular ultrastructural morphology in pancreatic acinar cells. In this study we focused on the impact of acute stress on acinar cells to provide a tool for investigation of the effect of acute peri‐transplant stress including organ preservation on early graft complications and loss [4, 9].

Acute changes including oedema, necrosis, leukocyte infiltration and acinar nodules have been observed in ischaemia studies in rodents and felines [33, 34, 35, 36, 37, 38, 39]. Reports of significant pathological changes (oedema, acinar necrosis and fat necrosis) associated with ischaemia in human pancreata assessed by light microscopy have largely been limited to experimental perfusion settings where the organ has been exposed to considerable *ex vivo* stress [21, 22, 23]. The current careful analysis of organs exposed to varying periods of ischaemia by an experienced pathologist, initially blinded to donor characteristics, supports the conclusion that histological evaluation using standard light microscopy is not sufficiently sensitive to quantitatively measure the impact of tissue stress and (sub)cellular injury in the pancreas.

Previous studies have confirmed the potential for evaluating established pancreatic pathologies at a sub‐cellular level through TEM. These have included accumulation of lipid droplets or lipofuscin bodies with potential associations with increasing donor BMI and donor age respectively described [40, 41]. These changes were observed in the current study and were readily distinguishable from the acute pathologies quantified within the newly derived NPASS. TEM analysis has been used before to analyse the impact of hypoxia or other stressors in a wide range of cells and tissues with chromatin condensation, vacuolisation, mitochondrial swelling, and ER dilation constituting the most frequently reported features. To our knowledge, these categories have not been quantified in the context of acute stress during organ procurement.

Condensed nuclear chromatin has been visualised in response to acute stress in rat cardiomyocytes, human placenta, a murine macrophage‐like cell line, human pancreatic cancer cells, rat pancreas, and human isolated islets [42, 43, 44, 45, 46, 47, 48, 49, 50]. Induction of hypoxia in these cells and tissues was associated with expression of activated caspase and annexin apoptotic markers in addition to evidence of structural DNA damage [42, 44, 45, 46, 48].

Development of vacuoles in association with apoptosis through hypoxia with/without reoxygenation has been observed in human placenta and human pancreatic cancer cells [42, 43]. This feature of acute stress on cells was furthermore observed in rat pancreas tissue samples experiencing prolonged warm ischaemia [47].

Other signs of stress observed in previous studies have included changes to mitochondrial morphology in human pancreatic cancer cells, rat endocrine cells, and rat pancreas in combination with additional apoptotic and/or acute changes [42, 47, 49, 51].

A further marker of acute stress observed in previous studies was dilation of ER even after short periods of exposure to hypoxia in rat pancreas in combination with additional acute changes [47, 51].

In ischaemia/reperfusion models in rats and pigs, dilation of ER, mitochondrial damage and loss of zymogen granules were all observed following restoration of blood‐flow in addition to decreased ATP levels and oxygen consumption, a reduction of functional capillary density, and increased amylase concentrations overall indicating tissue damage [52, 53]. Furthermore, these characteristic changes could also be confirmed in human pancreatic allograft biopsies taken 5 h post‐transplantation [54].

Each of these parameters of sub‐cellular stress was observed and scored in the current study and the range of potential changes was used to define each of the individual scoring parameters constituting NPASS. A rigorous and consistent approach for defining each numerical point within the scores was refined using the Development cohort. Interestingly, our independently derived scoring parameters are closely reflected in a recently published study characterising acute changes within murine pancreas in response to chemically induced (streptozotocin) diabetes [50].

NPASS was validated in a separate cohort of eight DBD and eight DCD pancreata with a wide range of CIT. Differential impact of pancreatic organ donation on acute TEM changes in nucleus, mitochondria, ER and cellular vacuolisation was observed. Relatively low nuclear and vacuolisation scores were seen even after prolonged CIT with or without additional warm ischaemia; suggesting that normothermic metabolism is required for more severe chromatin clumping (e.g. through apoptotic programmed cell death) and for vacuolisation to become manifest [43, 44, 47]. Mitochondria, on the other hand, exhibited marked changes after even brief ischaemia. Mitochondrial sensitivity to the stresses of deceased organ donation and preservation has previously been noted, with early swelling in static cold preservation relative to immediately fixed pancreas observed in rodents [47, 50, 51]. In contrast to the relatively consistent NPASS nuclear, mitochondrial and vacuolisation values irrespective of widely differing donor parameters, the full range of ER scores from 0.74 to 2.70 was seen in the Validation cohort.

Although the Validation cohort was relatively small, there was a possible trend towards higher EM scores in the tail in comparison to head of pancreas, reaching statistical significance for nucleus score in DBD organs. Potential mechanisms underlying the differential impact remain obscure although it could be postulated that dual vascularisation of the pancreatic head from both the gastro‐duodenal and superior mesenteric arteries may provide some protection from *peri‐mortem* ischaemia.

A trend towards higher EM scores in DCD *versus* DBD pancreata was also observed across all parameters but this only reached significance for mitochondrial changes, potentially reflecting progressive degradation initiated through warm ischaemia. Whilst the impact of additional warm ischaemia during procurement of DCD organs was smaller than expected, DCD organs have not usually been exposed to the ‘cytokine storm’ following brainstem death which elicits tissue damage similar to changes seen during sepsis [1, 2, 3]. Our findings are in keeping with the potential for comparable outcomes to DBD pancreas transplants with appropriate selection of DCD organs [4, 7].

Increasing CIT in DBD organs was reflected by higher ER scores with the highest correlation in the tail, supporting the use of a quantitative score of ER dilation as a ‘gold standard’ measure of acute cellular stress in deceased donor pancreata. Although associations were not observed between DBD ischaemic time and the other NPASS parameters, near‐significant correlation of total score with CIT was found.

In the DCD Validation cohort, no association between any NPASS parameter and CIT was observed. Similarly, higher scores were not found in donors exposed to the additional ischaemia associated with hypoxic brain death or cardiopulmonary arrest. This lack of correlation using an unbiased scoring system highlights the complex multifactorial causes of acute (sub)cellular stress in organ donors, with potential additional impact of, e.g. administration of vasoactive drugs, which are known to impact pancreatic blood supply but were not included in the current analysis [55]. Evidence of sub‐cellular stress was sensitively detected by EM assessment, but could not be recognised by light microscopy with no correlating light microscopy changes observed, confirming the lower sensitivity of light microscopy [56].

Conclusions from the current study are limited by the relatively small number of pancreata, although congruent processing procedures in all organs in the Validation cohort is a strength. Differences in sex distribution with six organs from female DBD (75%) *versus* one organ from female DCD (12.5%) have to be taken into consideration, with previous investigations revealing worse graft survival in recipients of female donor kidney allografts [57]. The purpose of the current study was, however, to develop the score and confirm its utility and validity in a second cohort. Whilst we have reported hypothesis‐generating findings, the study was not powered to formally confirm or refute correlations. A second study is currently underway where pancreas tissue is systematically exposed to prolonged longitudinal CIT to analyse the development of the NPASS parameters for acute cellular stress.

As an assessment of fixed tissue pre‐transplantation, NPASS may not fully predict the impact of ischaemia/reperfusion injury following restoration of blood flow and oxygenation [33, 34, 53, 58, 59]. The time‐intensive nature of TEM sample preparation and image acquisition precludes the use of NPASS in a clinical transplantation setting, but it is ideally suited to retrospective analysis of pre‐transplant biopsies and evaluation of cellular stress in an experimental setting. The score will facilitate more detailed elucidation of the impact of cellular stress on pancreatic ultrastructure, potentially yielding a deeper understanding of donor risk factors and optimal donor/retrieved organ management. The NPASS system has been designed to enable implementation as an unbiased research tool and we are implementing quality assured training schemes to facilitate access. The score may also have a role in systematically assessing potential *peri‐mortem* and tissue handling stresses which may otherwise confound accurate interpretation of pre‐morbid pathology in clinical diagnostic and research tissue bank settings.

We are currently applying NPASS to score donor pancreata biopsied sequentially during a 4 °C incubation to better understand evolution of (sub)cellular changes during cold ischaemia. Degradomics analysis is being undertaken in parallel with the goal of providing a point of care test accurately quantifying acute cellular stress which will aid clinical decision regarding progression to transplantation informed by a measure which is meaningfully predictive of recipient outcomes. The present study was purposefully limited to acinar cells but further studies are also being undertaken to assess the impact of acute cell stress on the pancreatic endocrine compartment.

The current detailed histological and EM analysis of a wide range of donor pancreata indicates that tissue stress and damage cannot be accurately predicted using simple clinical parameters or sensitively detected by conventional light microscopy. Adopting an unbiased and iterative approach, a robust EM score for semi‐quantitative assessment of acute cellular stress in human pancreatic tissue has been developed and validated. The NPASS provides a new tool for systematic characterisation of (sub)cellular ultrastructural changes within the pancreas which will enable clearer understanding and meaningful comparison between findings across the spectrum of clinical and research settings.

## Author contributions statement

NK, ND, JAMS and WES formulated the research idea and designed the study. MHS, JD and ND acquired the biopsies. TD, KW, LE, LT and BW provided crucial feedback on EM image interpretation. NK, ND and YB acquired, analysed and/or interpreted data. DT, RP, JAMS and WES supervised or mentored the study. NK wrote the original draft of the manuscript. Each author contributed during manuscript drafting or revision.

## Data availability statement

The data that support the findings of this study are available from the corresponding author upon reasonable request. Training for NPASS is available; please contact the corresponding author.

## Supporting information


**Figure S1.** Example image of islet peliosis presented as blood filled cavities
**Table S1.** Development cohort donor demographics
**Table S2.** Brief pathological reports for the 16 pancreata in the Validation cohort
