# Supplementary material for: Development and validation of a quantitative electron microscopy score to assess acute cellular stress in the human exocrine pancreas
Source: J Pathol Clin Res. 2020 Nov 22;7(2):173–87. doi: 10.1002/cjp2.185 (PMC7869933; doi:10.1002/cjp2.185)
Supplement: Supplementary file 1 — Figure S1. Example image of islet peliosis presented as blood filled cavities Table S1. Development cohort donor demographics Table S2. Brief pathological reports for the 16 pancreata in the Validation cohort [file CJP2-7-173-s001.docx]

**Development and validation of a quantitative electron microscopy score to assess acute cellular stress in the human exocrine pancreas**

N Kattner, N Dyson *et al. J Pathol Clin Res* DOI: 10.1002/cjp2.185

# Supplementary material


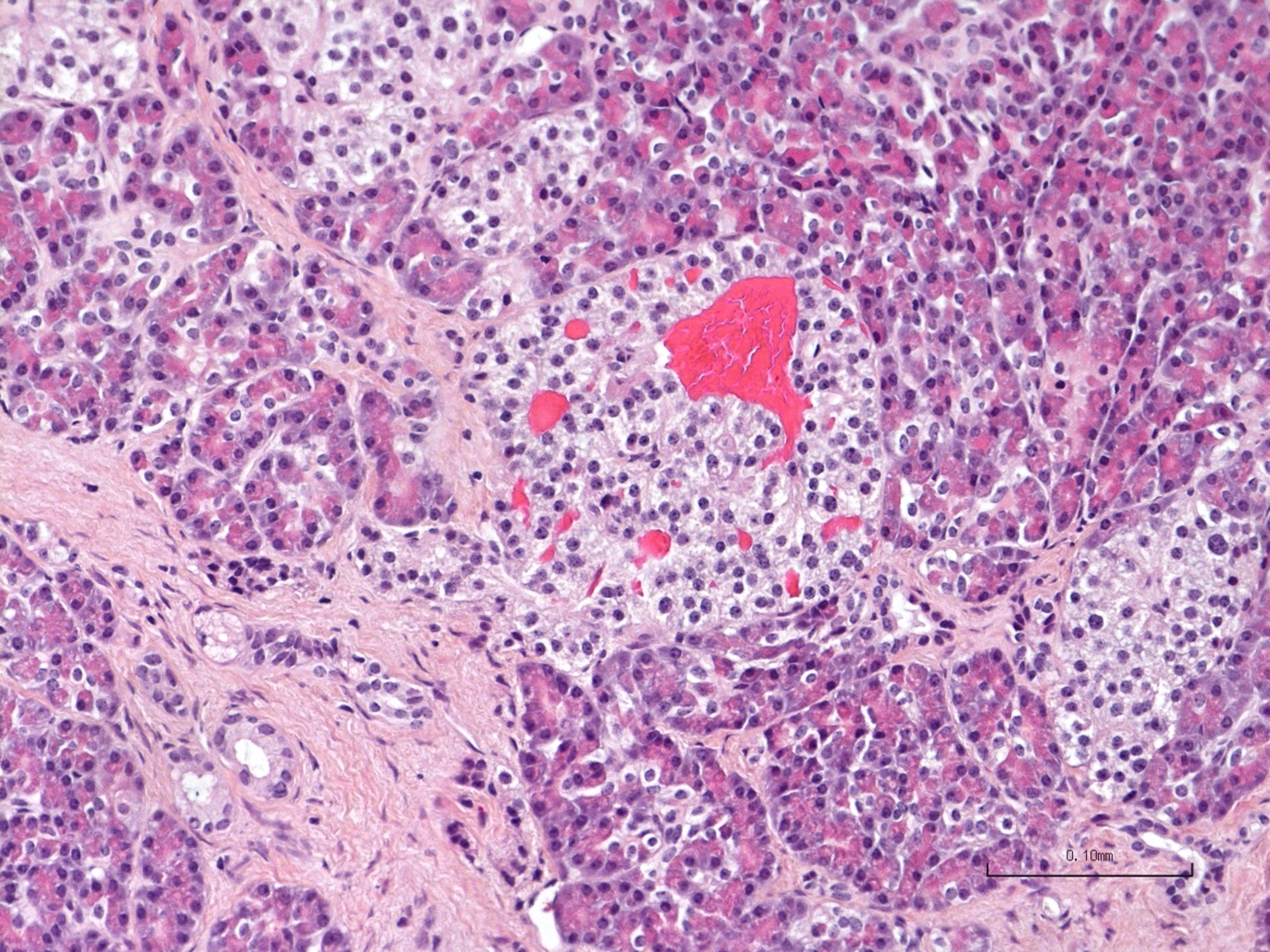


**Figure S1:** Example image of islet peliosis presented as blood filled cavities. (H&E staining of DBD-7 tail, x20).

**Table S1:** Development cohort donor demographics

| **Internal donor number** | **Sex** | **Age**  **(years)** | **BMI**  **(kg/m^2^)** | **Donor Type** | **CIT**  **(hours)** | **WIT**  **(mins)** | **Images in Figure 2** |
| --- | --- | --- | --- | --- | --- | --- | --- |
| 169 | female | 64 | 26.4 | DCD | 16.5 | 32 |  |
| 172 | male | 25 | 22.8 | DCD | 8.0 | 18 | B2, B3 |
| 173 | male | 43 | 26.8 | DBD | 5.1 | - | A1, A4, C1, C3 |
| 214 | male | 49 | 31.5 | DBD | 27.0 | - | B4 |
| 215 | male | 28 | 24.4 | DCD | 9.9 | 25 |  |
| 218 | male | 53 | 22.9 | DCD | 8.8 | 17 | A2, A3, B1, C4 |
| 219 | male | 39 | 28.3 | DCD | 29.8 | 29 | C2 |
| 221 | female | 57 | 23.2 | DBD | 38.1 | - |  |
| 226 | female | 39 | 24.3 | DBD | 18.0 | - |  |
| 231 | female | 65 | 17.7 | DCD | 6.4 | 34 |  |
| 232 | female | 73 | 24.8 | DCD | 8.9 | 33 |  |
| 237 | male | 69 | 29.1 | DBD | 6.3 |  |  |
| 244 | female | 49 | 26.6 | DCD | 13.2 | 12 |  |
| 253 | male | 67 | 22.9 | DBD | 4.2 | - |  |
| 254 | female | 48 | 33.4 | DBD | 8.4 | - |  |
| 255 | female | 70 | 34.9 | DBD | 7.0 | - |  |
| 257 | female | 63 | 31.0 | DBD | 8.1 | - |  |
| 258 | male | 63 | 26.0 | DBD | 22.0 | - |  |
| 259 | female | 28 | 26.8 | DBD | 18.2 | - |  |
| 260 | female | 40 | 31.6 | DBD | 22.2 | - |  |
| 263 | female | 63 | 32.8 | DBD | 5.0 | - |  |
| 264 | male | 38 | 23.2 | DBD | 4.7 | - |  |
| 265 | female | 60 | 23.6 | DCD | 5.9 | n/s |  |
| 267 | female | 58 | 21.4 | DBD | 9.1 | - |  |
| 269 | male | 62 | 28.7 | DBD | 22.2 | - |  |
| 270 | female | 33 | 21.6 | DBD | 6.5 | - |  |
| 271 | male | 74 | 30.5 | DCD | 12.3 | n/s |  |
| 272 | male | 59 | 27.2 | DCD | 10.9 | n/s |  |
| *Mean* |  | *53* | *27* |  | *12.9* | *25* |  |
| *Range* |  | *25-74* | *17.7-34.9* |  | *4.2-38.1* | *12-34* |  |
| *Summary* | *15 (54%) female* |  |  | *17 (61%) DBD* |  |  |  |

Donor demographics including internal donor ID number, sex, age, body mass index (BMI), cold ischaemia time (CIT), warm ischaemia time (WIT), and associated images in Figure 2.

**Table S2:** Brief pathological reports for the 16 pancreata in the Validation cohort (summarised in Table 3)

|  | **Morphological assessment with light microscopy (H&E and SRFG sections)** | |
| --- | --- | --- |
| **Donor number** | **Head** | **Tail** |
| **DBD-1** | No significant changes, minimal intralobular fibrosis. Scattered fat cells in lobules. Rare eosinophilic acinar cell nodules. Good quality section. No significant inflammation, no significant necrosis or marked morphological features of ischaemia. | No significant changes, minimal intralobular fibrosis. Rare eosinophilic acinar cell nodules. Slight intra parenchymal lipomatosis, slightly more pronounced than in head. Good quality section. No significant inflammation, no significant necrosis or marked morphological features of ischaemia. |
| **DBD-2** | No significant changes, negligible fibrosis, no significant fat necrosis. Focal lipomatous change. Rare and very mild acinar luminal ectasia with secretions. Very minimal discohesion focally. Good quality section. No significant inflammation, no marked morphological features of ischaemia. | Very focal acinar luminal ectasia with secretions. Very focally degenerative changes of intercalated ducts. Good quality section. No significant inflammation, no significant necrosis and no significant features of ischaemia. |
| **DBD-3** | Moderate lipomatosis with focal periductal and intralobular fibrosis. Features of atrophy in acinar cells. Focal islet peliosis. Some minor focal acinar discohesion. Good quality section. No significant inflammation, no significant necrosis, no marked morphological features of ischaemia. | Significant periductal and intralobular fibrosis with associated atrophy, affecting approximately half of the tissue. Focal degenerative vascular changes with hyaline arteriosclerosis. Good quality section. No significant inflammation, no significant necrosis, no marked morphological features of ischaemia. |
| **DBD-4** | Minor changes with minimal focal periductal and intralobular fibrosis. Low grade PanIN (1A) in branch duct. Good quality section. No significant inflammation, no significant necrosis or marked morphological features of ischaemia, although focally slight separation / discohesion of acini. Acinar cell cytoplasm in some areas slightly more basophilic. | Minor changes with minimal focal periductal and intralobular fibrosis. One hyperplastic islet. Good quality section. No significant inflammation, no significant necrosis or marked morphological features of ischaemia. |
| **DBD-5** | Mild to moderate periductal and intralobular fibrosis. Good quality section. Focal discohesion of acini. No significant inflammation, no significant necrosis or marked morphological features of ischaemia. | Mild to moderate periductal and intralobular fibrosis. Minor discohesion of acini. Good quality section. No significant inflammation, no significant necrosis or marked morphological features of ischaemia. |
| **DBD-6** | Focal minor changes with mild focal periductal and intralobular fibrosis. Few larger hyperplastic islets. Minimal focal discohesion between acini. No significant inflammation, no significant necrosis or marked morphological features of ischaemia. | Mild to moderate mainly perilobular fibrosis with some periductal and intralobular fibrosis. One islet congested with peliosis. Focal discohesion between acini. One area with lymphocytic inflammation. No significant necrosis or marked morphological features of ischaemia. |
| **DBD-7** | Overall mild focal changes with mild focal periductal and intralobular fibrosis and focal mild atrophy, most pronounced in one small lobule. Good quality section. Very focal discohesion between acini. No significant inflammation, no significant necrosis or marked morphological features of ischaemia. | Overall mild focal changes with mild focal periductal and intralobular fibrosis associated with very mild/minor focal atrophy. Low grade PanIN in main and adjacent branch duct. Good quality section, no significant inflammation, no significant necrosis or marked morphological features of ischaemia. |
| **DBD-8** | Focal mild changes with intralobular fibrosis. Low grade PanIN in main duct. Very focal atrophy at edge of the section. Very focal intralobular single cell fat necrosis. Good quality section. No significant inflammation, no significant necrosis or marked morphological features of ischaemia. | Significant fibrosis with marked atrophy with islet aggregation and islet hyperplasia, affecting approximately one third to half of the tissue section. Low grade PanIN in main and branch ducts. Good quality section. No significant inflammation, no significant necrosis or marked morphological features of ischaemia. |
| **DCD-1** | Moderate to severe lipomatosis/fatty change. Scattered eosinophilic acinar cell nodules (n=15) and also few foci of acinar cells with inclusion bodies (n=6). Focal islet hyperplasia. Minimal fibrosis. Relatively prominent fat content in the lobules. One area of inflammation or benign vascular proliferation. Otherwise good quality section. Minimal focal chronic inflammation, but no significant necrosis or marked morphological features of ischaemia. | Mild periductal fibrosis with acinar ductal and squamous metaplasia. Numerous eosinophilic acinar cell nodules (n=14) and foci of acinar cells with inclusion bodies (n=3), which are more prominent than in block of head area. (= Foci of intracytoplasmic globules similar to benign ground glass inclusions or inspissated protein / mucin in few acinar cell groups. Advice sought from expert pancreas pathologists BH and GK) Scattered fat cells in the lobules. Good quality section. No significant inflammation, no significant necrosis or marked morphological features of ischaemia. |
| **DCD-2** | Very minor changes with minimal focal periductal and intralobular fibrosis. Very minor focal discohesion of acini. One tiny focus of eosinophilic pale acinar cells but too small for unequivocal proper acinar cell nodule. One hyperplastic islet. Good quality, no significant inflammation, no significant necrosis or marked morphological features of ischaemia. | Mild changes with minimal focal periductal and intralobular fibrosis. Low-grade PanIN in part of the main duct. Very minor focal discohesion of acini. Good quality section, no significant inflammation, no significant necrosis or marked morphological features of ischaemia. |
| **DCD-3** | 2.5 mm focus of intraductal proliferation, in this area more in keeping with low-grade PanIN than IPMN (too small in this section - confirmed by second specialist pancreatic expert pathologist BH). Significant periductal and perilobular fibrosis with focal atrophy and acinar ductal metaplasia. Good quality section. Minimal focal chronic inflammation, but no significant necrosis or marked morphological features of ischaemia. | End stage atrophy with few residual islets and no convincing exocrine cells and scattered lymphoid aggregates, focally suggestive of incipient small lymph nodes. Technically good quality section. No significant features of necrosis or ischaemia. |
| **DCD-4** | Significant periductal and intralobular fibrosis with associated atrophy. Focal islet peliosis / islet congestion and islet hyperplasia. Focal inspissated secretions in main duct with mild focal acute inflammation with neutrophil polymorphs. Focal nesidioblastosis (islet/duct complexes). Focal inflammation, but no significant necrosis or marked morphological features of ischaemia. Good quality section. | Significant fibrosis with marked atrophy with islet aggregation and islet hyperplasia, affecting approximately at least half of the tissue section. Focal islet peliosis / congestion. Suggestion of focal minimal low grade PanIN in main duct. No significant inflammation, no significant necrosis or marked morphological features of ischaemia. |
| **DCD-5** | Minor changes with minimal focal perilobular fibrosis. Focal intraluminal secretions in one small branch duct. Focally slightly hyperplastic islets. In one area poor cohesion of exocrine acini and features suggestive of possible early ischaemic change or fat necrosis, but tissue lifting in this area. Otherwise good quality section. No significant inflammation, no significant necrosis or marked morphological features of ischaemia. Scattered fat cells within parenchyma. | No significant changes with minimal focal periductal fibrosis at most. Very mild focal squamous metaplasia of main duct. Focal minimal intracellular steatosis in islets cells in one islet. Focal discohesion of acini. Good quality section. No significant inflammation, no significant necrosis or marked morphological features of ischaemia. Scattered fat cells in parenchyma marginally less than in head. |
| **DCD-6** | Mild focal periductal and perilobular fibrosis. Focal interlobular oedema. Focal squamous metaplasia of branch ducts. Focal minimal acinar ectasia. Good quality section. No significant inflammation, no significant necrosis or marked morphological features of ischaemia. | Minimal focal periductal and intralobular fibrosis. Focal single cell fat necrosis and minimal acinar cell necrosis. Focal minimal acinar ectasia. Focal islet cell hyperplasia. Good quality section. No significant inflammation, minimal focal necrosis, but no marked morphological features of ischaemia. |
| **DCD-7** | Focal periductal fibrosis. Focal degenerative vascular changes with arteriosclerosis. Focal islet hyperplasia. Focal discohesion of acini. Good quality section. No significant inflammation, no significant necrosis, no marked morphological features of ischaemia. | Minimal periductal fibrosis. Focal islet hyperplasia. Focal discohesion of acini could be artefact or early ischaemia. Good quality section. No significant inflammation, no significant necrosis, no marked morphological features of ischaemia. |
| **DCD-8** | No significant changes with minimal focal perilobular fibrosis. Very minimal focal fat necrosis and peripancreatic adipose tissue. Minimal mucinous metaplasia in branch duct, not amounting to low-grade PanIN. Good quality section. Scattered fat cells in the lobules. No significant inflammation, no significant necrosis or marked morphological features of ischaemia. | No significant changes with minimal focal perilobular fibrosis. Very focal islet peliosis. Minimal single cell fat necrosis in intra/peripancreatic adipose tissue. Good quality section. No significant inflammation, no significant necrosis or marked morphological features of ischaemia. |
